# Ethnobotanical perspective of antimalarial plants: traditional knowledge based study

**DOI:** 10.1186/s13104-015-1827-z

**Published:** 2016-02-04

**Authors:** Abdul Qayum, Rakesh Arya, Andrew M. Lynn

**Affiliations:** Center for Biology and Bioinformatics, School of Computational and Integrative Sciences, Jawaharlal Nehru University, New Delhi, 110067 India; Centre for the Study of Regional Development, Jawaharlal Nehru University, New Delhi, India; Indira Gandhi National Forest Academy, Dehradun, India

**Keywords:** Antimalarial plants, Ethnobotany, Ethnoecology, Geographical health, Medicinal plants, Plant indices, Traditional knowledge system

## Abstract

**Background:**

Considering the demand of antimalarial plants it has become essential to find and locate them for their optimal extraction. The work aims to find plants with antimalarial activities which were used by the local people; to raise the value of traditional knowledge system (TKS) prevalent in the study region; to compile characteristics of local plants used in malaria treatment (referred as antimalarial plants) and to have its spatial distribution analysis to establish a concept of geographical health.

**Methods:**

Antimalarial plants are listed based on literature survey and field data collected during rainy season, from 85 respondents comprised of different ethnic groups. Ethno-medicinal utilities of plants was extracted; botanical name, family, local name, part used, folklore, geographical location and image of plants were recorded after cross validating with existing literatures. The interview was trifurcated in field, *Vaidya/Hakims* and house to house. Graphical analysis was done for major plants families, plant part used, response of people and patients and folklore. Mathematical analysis was done for interviewee’s response, methods of plant identification and people’s preferences of TKS through three plant indices.

**Results:**

Fifty-one plants belonging to 27 families were reported with its geographical attributes. It is found plant root (31.75 %) is used mostly for malaria treatment and administration mode is decoction (41.2 %) mainly. The study area has dominance of plants of family Fabaceae (7), Asteraceae (4), Acanthaceae (4) and Amaranthaceae (4). Most popular plants found are *Adhatoda vasica, Cassia fistula* and *Swertia chirata* while  % usage of TKS is 82.0 % for malaria cure.

**Conclusion:**

The research findings can be used by both scientific community and common rural people for bio-discovery of these natural resources sustainably. The former can extract the tables to obtain a suitable plant towards finding a suitable lead molecule in a drug discovery project; while the latter can meet their local demands of malaria, scientifically.

**Electronic supplementary material:**

The online version of this article (doi:10.1186/s13104-015-1827-z) contains supplementary material, which is available to authorized users.

## Background

Traditional medicine plays a pivotal role in the economy and sustainable growth of developing nations especially for poor countries like India. The preventive health measures have still not been able to penetrate the economically downtrodden societies including typical rural and schedule tribe areas. Their life is purely dependent on traditional knowledge system (TKS) of herbal plants for medicinal cure purposes. The native people are exploiting a variety of herbals for effective curing of various ailments and most widely malaria. Such herbal plants possess potential remedy even for some incurable diseases as well. The plant parts used, preparation method of medicine from it, and its administration as drug varies geographically [1]. However, the knowledge of herbal medicines is gradually perishing, although some of the traditional herbal men like Vaidyas and Hakims are still practicing the art of herbal healing effectively in such regions. Ethno-medicinal and ethno-botanical studies have offered immense scope and opportunities for the development and synthesis of new drugs. Modern drugs have been deducted from folklore and traditional medicines [2].

Malaria is a culprit that has victimized almost half of the modern civilization and it is endemic across more than 100 countries [3]. Malaria is a major public health problem in India, 40 million people are suffering from this single celled *Plasmodium*, a protozoan parasite and more than 1.5 million confirmed cases are reported annually by the National Vector Borne Disease Control Programme (NVBDCP), New Delhi of which 40–50 % is due to *Plasmodium falciparum* (Pf). However, in the study area malaria is only due to *Plasmodium vivax* as Pf count was found to be zero [4]. Malaria can be considered as poor man’s disease and hence it requires focussed planning both at the level of government and at the community level.

In the *Tarai* regions of Eastern Uttar Pradesh (UP) the spreading of vector borne diseases becomes uncontrolled especially during rainy seasons [4]. In recent studies, chloroquine-resistant *P. falciparum* malaria has been observed with increasing incidence in the whole country, while it was effective for treating nearly all cases of malaria in the past. *P. falciparum* has become drug resistant now. The continued treatment of such cases with chloroquine is probably one of the factors responsible for the increased proportion of *P. falciparum* cases relative to *P. vivax* [5]. The current malaria therapies include methods such as passive surveillance of malaria, use of artemisinin combination therapy (ACT) and introduction of intervention like rapid diagnostic tests (RDT) for malaria cure [6]. The commercial industry on antimalarial drug is facing huge a challenge primarily due to development of drug resistance of malarial parasites. And, hence bio-discovery of antimalarial plants has become inevitable towards discovering a novel plant in order to find a lead compound towards malaria medication efforts. It is very likely that in times to come sustainable harvesting of this natural wealth needs to be done to meet the public health demands.

In this circumstance, ethnobotanical and ethnoecological study of antimalarial plants in three districts of Eastern UP was carried out. The native communities have been using their unique traditional knowledge (TK) system, culture, indigenous skills and expertise since the ancient times towards the disease control. TK refers to the ancient and non-conventional practices towards disease control mechanism. Local knowledge of a community is spread across various diseases and masses from developing countries utterly rely on the herbal treatment methods. India has witnessed its legacy from the times of Charaka and Susruta for traditional knowledge for antimalarial activities of various medicinal plants [7]. Considering the high time demand of antimalarial plant it has become inevitable to find and locate them for its optimal extraction for antimalarial actions.

A series of similar work has been done by many people in the country including Verma et al. [2] that has found 72 plant species in campus of Banares Hindu University, Varanasi, UP and has compiled the traditional uses by the local inhabitants. Ethnobotanical work has also been done for the Gorakhpur regions [8, 9] while Kumar and Akhtar [10] have worked on ethanomedicinal solanaceous plants of eastern UP and have found 14 species of medicinal angiosperms. Qayum et al. [1] has done geographic information system based study for antimalarial plants in three districts of UP to highlight geographical attributes of these plants. Tomar [11] has worked on folk medicinal uses of plant roots of Meerut city and has found 39 medicinal plant species belonging to 39 genera and 28 families which are used by rural and common people for various diseases. Srivastava [12] has done ethnobotanical exploration with respect to food values of 27 underutilized edible fruits consumed by ethnic people of north-eastern *terai* region of UP and have established database of these fruits which is useful in bio-discovery projects for achieving food security and environmental sustainability. Shankar et al. [13] have listed all the antimalarial plants of north east India and has emphasized the need for an alternative drug for malaria for developing new antimalarial plants from the indigenous plants.

Panda et al. [14] have documented phyto-therapeutical practices in Mayurbhanj district of Orissa, eastern India and has described 112 plants from 62 families which are therapeutically used against different ailments including malaria. Bahekar and Kale [15] on “Herbal Plants Used for the Treatment of Malaria” have highlighted many plants which are used since ancient times for the treatment of malaria and have established pharmacotherapy is the most common treatment strategy for the disease. Sampath et al. [16] have described *Swertia chirata* as a traditional herb and has found it is used as a preventative measure for malaria during epidemics. Kamaraj et al. [17] has done an ethno-pharmacological investigation on antimalarial activities of medicinal plants traditionally used in the villages of Dharmapuri regions of south India and has identified 24 such plants.

The research objectives are to find all possible plants in the study area with antimalarial activities which were used by the local people and the tribes. This was intended to know and appreciate the TK system based medication prevalent in the region. Therefore, it is aimed to compile and list characteristics of antimalarial plants prevalent in three districts of Kushinagar, Maharajganj and Gorakhpur of eastern UP and to show the distribution of antimalarial in various geographical regions. Further, the scope of present work has been extended to find out geographical health (GH) of antimalarial plants. GH refers to the state of plant presence in the study area. It is found that many plants are at the verge of either extinction, critically endangered or vulnerable [18]. Hence, immediate attention is highly expected from policy makers to safeguard the interest of people at large and some immediate action is needed from the government bodies to safeguard the current bio-diversity and medicinal plant richness of the region.

The current work is a fine mixture of literature survey and supplemented with collected information through interviews and field visits after cross validating the study area findings from *Vaidyas/Hakims*, and native rural people. The TK skills and practices thus developed are freely exchanged, cared for and nourished as a common property of the communities [19]. The current work has also highlighted the geographical location of antimalarial plants along with listing them with its family name, folklore, and image of plant in its natural habitat and geographical health has also been explored. It is anticipated that with these observations; traditional method of malaria cure can be accelerated especially for the low socio-economic regions where modern health measures are nearly absent.

## Methods

### Study area

**Fig. 1:**
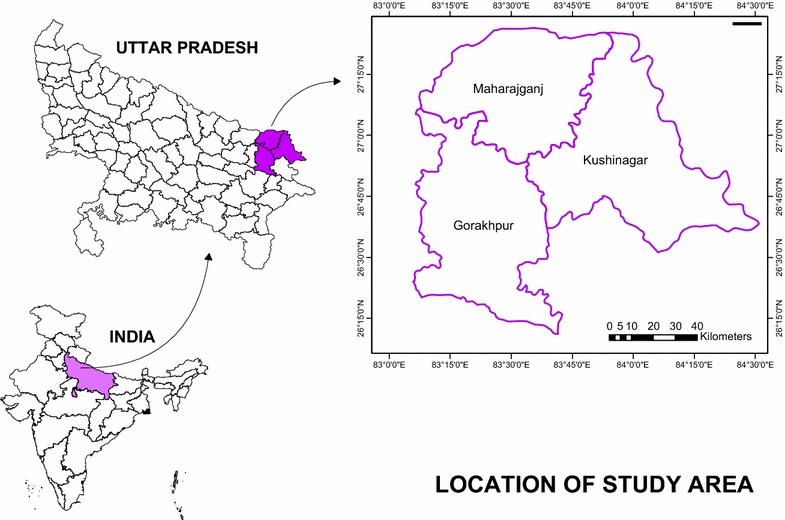
Location of study area

The study area lies in the north-eastern corner of the most populous state of UP, India. It comprises a large stretch lying to the north of the river Rapti tributary to the Gandak river and also surrounded by Rohini river at northern side which is the major source of water in the region. The area comprised of three district of UP viz. Gorakhpur, Kushinagar and Maharajganj (Fig. 1). It has total area of 9291 Km^2^ (3.82 % of the state). This much of land area is home to people of countries like, Greece, Portugal, Sweden, etc. It also shares international borders with Nepal. The study area is one among highly dense region of the country (average density around 1210 people per Km^2^) and is home to more than 10.67 million Indian population [20]. The region is one among several vector borne diseases sensitive zones especially for Japanese encephalitis and malaria.

The study region is home to Tharu, Buksa, Raji and Jaunsari tribes (114,876 people) which are 1.075 % of total population. Two religious communities Hindus (82 %) and Muslims (15 %) find dominance while Buddhists and others constitute 3 % population of the study area. The economics is primarily based on agriculture (97.48 %) with 87,400 main industry working and to some extent on tourism because of presence of Kushinagar Baudh temple (one of the main attraction for the Buddhists). As per the recent census (2011) of UP, India [20] total Population of study area is 10,690,142 (Table 1) with 48.60 % female population and 1,680,587 as total house hold of which 87.98 % is rural house hold. The rural people migrate to big cities for work very often. Entire study area happens to be least developed part of one of the poorest State in India. It makes low socio-economic profile for the region with 3,462,855 total work participation (female: 28.3 %) and 1,708,932 main work participation (female: 19.1 %) and 1,753,923 marginal worker (female: 37.3 %) (Fig. 1).

**Table 1:** Socio-economic profile of the study area

District name	Population type	Total house hold	Total population	Total male	Total female
Gorakhpur	Rural	554,999	3,604,766	1,838,726	1,766,040
	Urban	137,961	836,129	439,051	397,078
	Total	692,960	4,440,895	2,277,777	2,163,118
Kushinagar	Rural	533,834	3,396,437	1,730,377	1,666,060
	Urban	27,228	168,107	87,678	80,429
	Total	561,062	3,564,544	1,818,055	1,746,489
Maharajganj	Rural	175,359	1,101,460	564,281	537,179
	Urban	7497	45,201	23,349	21,852
	Total	182,856	1,146,661	587,630	559,031

Source: census of India, 2011

### Methodology

**Fig. 2:**
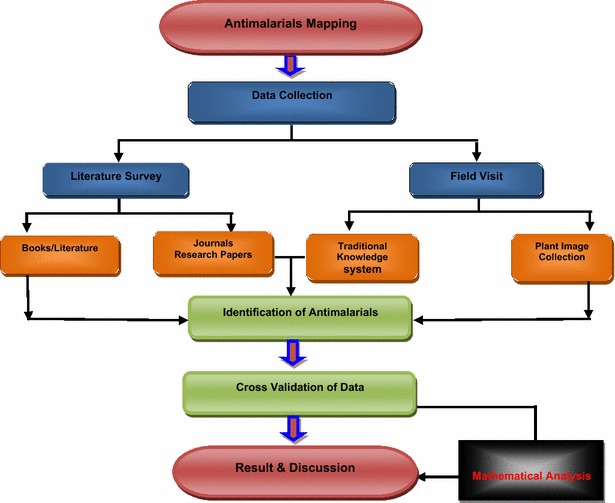
Schematic flowchart for the identification of antimalarial plants

The work has been carried out as per the schematic flowchart (Fig. 2) and its various dimensions are

### Pre-ethnobotanical surveys

Before conducting actual ethnobotanical survey pre-ethnobotanical survey was conducted to set the standard goal targets. It began with extraction of ethno-medicinal utilities of plants extracted from the relevant literature available in journals and books and cross examined with accessing traditional knowledge (TK) available with local community including tribal. Numerous related publications of different journals, accepted literature from books like Flora of upper Gangetic Plains [21], Flora Gorakhpurensis [22], Herbal Medicines [23] were searched and cross examined with databases like PubMed Central (PMC) and finally this collected information was compared with the field data collected.

### Ethnobotanical survey setup

Survey was conducted in the study area based on the interviews of respondents chosen on criteria discussed below. The interview was trifurcated with respect to field, *Vaidya/Hakims* and house to house (Table 2). Total of 51 plants (Table 3) were recorded and tabulated. Interview was conducted to find out number of positive cases (n) towards malaria treatment for a particular plant of the region.

**Table 2:** Methods of plant identification and people’s preferences

Plant species	Families	Method of interview			Positive response (n)	PK (%)	PR	PI^a^
		Field (√)	Vaidya/hakim (*)	House to house (#)				
*Abrus precatorius* Linn.	Fabaceae			#	26	30.6	3	Fair
*Acacia farnesiana* (L.) Willd.	Fabaceae	√			0	0	3	Unknown
*Achyranthes aspera* Linn.	Amaranthaceae	√	*		30	35.1	1	Fair
*Adhatoda vasica* Nees	Acanthaceae	√	*	#	49	57.3	1	Good
*Aerva lanata* (L.) Juss. ex Schult.	Amaranthaceae	√		#	6	7.1	2	Poor
*Alstonia scholaris* (L.) R.Br.	Apocyanaceae		*		29	34.1	1	Fair
*Alternanthera sessilis* (L.) R.Br. exDC.	Amaranthaceae			#	6	7.1	2	Poor
*Amaranthus spinosus* Linn.	Amaranthaceae	√			4	4.7	3	Poor
*Ammannia baccifera* Linn.	Lythraceae	√			0	0	3	Unknown
*Andrographis paniculata* Wall. ex Nees	Acanthaceae	√	*	#	46	53.8	1	Good
*Asteracantha longifolia* (L.) Nees	Acanthaceae			#	8	9.4	3	Poor
Azardirachta indica A.Juss	Meliaceae	√		#	27	31.8	1	Fair
*Barleria prionitis* Linn.	Acanthaceae			#	12	14.1	2	Poor
*Bauhinia variegata* Linn.	Fabaceae	√	*	#	43	50.3	1	Good
*Boerhaavia diffusa* L.nom.cons.	Nyctaginaceae		*		26	30.6	1	Fair
*Caesalpinia crista* (Linn.)	Fabaceae		*		28	32.9	2	Fair
*Carica papaya* Linn.	Caricaceae	√	*	#	45	52.7	1	Good
*Cassia fistula* Linn.	Fabaceae	√	*	#	51	59.7	1	Good
*Cissampelos pareira* Linn.	Menispermaceae	√			0	0	3	Unknown
*Citrus medica* Linn.	Rutaceae			#	11	12.9	2	Poor
*Clerodendron infortunatum* Linn.	Verbenaceae	√		#	9	10.6	2	Poor
*Cosmos sulphureus* Cav.	Asteraceae	√			0	0	3	Unknown
*Cuscuta reflexa* Roxb.	Convolvulaceae		*		32	37.6	1	Fair
*Cyperus scariosus* Br.	Cyperaceae		*		28	32.9	2	Fair
*Datura metel* Linn.	Solanaceae	√			11	12.9	2	Poor
*Eclipta prostrata* (Linn.)Linn	Asteraceae	√		#	17	20.0	1	Poor
*Erythrina variegata* Linn.	Fabaceae	√			0	0	3	Unknown
*Jatropha gossypifolia* Linn.	Euphorbiaceae	√			0	0	3	Unknown
*Lantana camara* Linn	Verbenaceae	√		#	13	15.3	1	Poor
*Leucas aspera* (Willd.) Link.	Lamiaceae			#	26	30.6	2	Fair
*Ludwigia octovalvis* (Jacq.) P.H. Raven	Onagraceae			#	7	8.2	3	Poor
*Magnolia grandiflora* Linn.	Magnoliaceae	√			0	0	3	Unknown
*Momordica charantia* Linn.	Cucurbitaceae			#	3	3.5	3	Poor
*Murraya koenigii* (L.) Sprengel	Rutaceae	√			8	9.4	2	Poor
*Nyctanthes arbor*-*tristis* Linn.	Oleaceae		*		10	11.8	1	Poor
*Ocimum sanctum* Linn.	Lamiaceae			#	28	32.9	1	Fair
*Oenanthe javanica* (BL.) DC.	Apiaceae	√		#	13	15.3	2	Poor
*Piper longum* Linn.	Piperaceae		*	#	30	35.1	1	Fair
*Pongamia pinnata* (L.) Pierre	Fabaceae		*	#	45	52.7	1	Good
*Putranjiva roxburghii* (Wall.)	Euphorbiaceae	√		#	27	31.8	2	Fair
*Rauvolfia serpentine* (Linn.) Benth.	Apocynaceae	√			4	4.7	3	Poor
*Scoparia dulcis* Linn.	Scrophulariaceae	√			0	0	2	Unknown
*Sida rhombifolia* Linn.	Malvaceae	√		#	5	5.9	2	Poor
*Solanum indicum* Linn.	Solanaceae	√	*		12	14.1	1	Poor
*Stephania japonica* (Thunb.) Miers	Menispermaceae	√			3	3.5	3	Poor
*Streblus asper* Lour.	Moraceae	√			0	0	3	Unknown
*Swertia chirata* Buch.Ham	Gentianaceae	√	*	#	48	56.2	1	Good
*Tinospora cordifolia* (Thunb.) Miers.	Menispermaceae	√	*	#	43	50.3	1	Good
*Vandellia sessiliflora* Benth.	Scrophulariaceae	√			0	0	3	Unknown
*Vernonia cinerea* (Linn.) Less.	Asteraceae	√		#	0	0	3	Unknown
*Xanthium strumarium* Linn.	Asteraceae	√	*		20	23.5	2	Poor

^a^PI is ‘Good’ if PK ≥ 50 %, ‘Fair’ if 30 % ≤ PK < 50 %, ‘Poor’ if 0 < PK < 30 %, and ‘Unknown’ if PK = 0 %

**Table 3:** List of plants used for malaria treatment in the study region

S.no	*Botanical name* (Voucher no)	Family	Vernacular/local name	Part used	Method used for cure	Plant location	Reference	Plant image
1.	*Abrus precatorius* Linn. 10787	Fabaceae	Ratti, Ghumchi	Whole plant	Two spoonful decoction of plant is taken orally twice a day for 3 days	Fruit orchard and climbing over trees	[14], [22]: p. 89	
2.	*Acacia farnesiana* (L.) Willd. 10788	Fabaceae	Tarua kadam (Ass)	*Bark*	^a^Decoction of bark is used	Found wild in wastelands, cultivated in gardens	[13], [22]: p. 125, [25]	
3.	*Achyranthes aspera* Linn. 10789	Amaranthaceae	Chirchita/ Apamarga/ Chitchita	Roots	With kali mirch used for intermittent fever and malaria	Roadsides, hills up to 900 m, Railway lines, wastelands	[22]: p. 271, [26]: p. 39, [27]: p. 2066	
4.	*Adhatoda vasica* Nees 10790	Acanthaceae	Arusa/ Vasaka	Roots	Powdered roots are used by native doctors for malaria	Cultivated lands, Waste places and Railway tracks	[22]: p. 240, [27]: p. 1899	
5.	*Aerva lanata* (L.) Juss. ex Schult. 10791	Amaranthaceae	Gorakhganja/ Bhadra	Whole plant	Plant is grinded and mixed with water and given to patient	Weed of crop fields, in fruit orchards	[2], [22]: p. 271, [26]: p. 67	
6.	*Alstonia scholaris* (L.) R.Br. 10792	Apocyanaceae	Saptaparna/ Saptachada/ chatiyan	Leaves, Bark and Flower	Decoction is prepared and 2 teaspoon is given twice a day with honey	Planted as roadside tree and also found wild	[2], [22]: p. 192, [26]: p. 111	
7.	*Alternanthera sessilis* (L.) R.Br. ex DC. 10793	Amaranthaceae	Gudrisag/ Matsyaksi	Leaves and Stems	Decoction of leaf is given	Weed of cultivated field and in moist waste places	[1], [22]: p. 273, [26]: p. 118	
8.	*Amaranthus* *spinosus* Linn. 10794	Amaranthaceae	Chaulai Bhaji, Cholai	Roots	Juice obtained from squeezed roots is mixed with powdered rice and taken with water till cure	Along roads, canals, railway tracks and a weed of cultivated fields	[22]: p. 274, [28]	
9.	*Ammannia baccifera* Linn. 10795	Lythraceae	Dadamari	Leaves	Leaves are used in fever	Marshy lands, Paddy fields, moist places	[22]: p. 136, [26]: p. 125, [35]	
10.	*Andrographis paniculata* Wall. ex Nees 10796	*Acanthaceae*	Kalmegh	Leaves	Decoction of leaves is taken orally for twice a day with half glass of milk	Shady waste grounds	[14], [22]: p. 241	
11.	*Asteracantha longifolia* (L.) Nees 10797	Acanthaceae	Talmakhana	Whole plant	Juice of whole plant is given to patients	Shallow ditches along roads	[4], [22]: p. 244	
12.	*Azardirachta indica* A.Juss 10798	Meliaceae	Neem	Leaves and Fruits	Decoction of leaves and fruits is given	Planted in gardens and near temples	[2], [22]: p. 70, [26]: p. 227	
13.	*Barleria prionitis* Linn. 10799	Acanthaceae	Kastira, Bajradanti	Leaves	Decoction of leaf is given with honey for 7 days	Waste ground, planted as border plant	[14], [22]: p. 242	
14.	*Bauhinia variegata* Linn. 10800	Fabaceae	Kachnar	Bark, Root and Leaves	Decoction of bark, root and/or leaves is used	Planted in gardens and as roadside tree	[2], [22]: p. 115, [26]: p. 256	
15.	*Boerhaavia diffusa* L.nom.cons. 10801	Nyctaginaceae	Gadapurna / Punarnava	Roots	Drink this herb root paste for malaria treatment	Elevated lands, roadsides, railway tracks, waste places, crevices of walls and fruit orchards	[22]: p. 269, [26]: p. 281, [29]	
16.	*Caesalpinia crista* (Linn.) 10802	Fabaceae	Kat-karanj, Karanju	Roots and Seeds	Prepared in form of dry powder in dose of ½ gm with honey	Open wastelands and along Nallas	[2], [22]: p. 115, [26]: p. 320	
17.	*Carica papaya* Linn. 10803	*Caricaceae*	Papita	Leaves	Decoction of leaves is used	Cultivated around bungalows and gardens	[22]: p. 143, [25]	
18.	*Cassia fistula* Linn. 10804	Fabaceae	Amaltas	Fruits and Buds	Decoction of fruits and buds are used	Deciduous tree Planted roadside and in gardens	[22]: p. 118, [30]: p. 856	
19.	*Cissampelos pareira* Linn. 10805	Menisperma-ceae	Harjuri, Bharat-buti	Root	Juice of the root is administered	Fruit orchard, and as hedges of parks and gardens	[22]: p. 34,	
20.	*Citrus medica* Linn. 10806	Rutaceae	Jameri-nimbu	Fruit	Juice is of fruit is used	Planted in Gardens, Lahladpur area	[22]: p. 67, [25]	
21.	*Clerodendron infortunatum* Linn. 10807	Verbenaceae	Bhat	Root and Leaves	One tea spoonful leaf juice is taken 3 times daily for a week	Under shades of trees, fruit orchards	[22]: p. 254, [28], [31]	
22.	*Cosmos sulphureus* Cav. 10808	Asteraceae	Cosmos	Leaves	Leaves and aerial are part used in intermittent fever	Throughout study area	[9]	
23.	*Cuscuta reflexa* Roxb. 10809	Convolvulaceae	Amarbel	Stem	Paste of about 10 gm stem and 7 black pepper seeds is taken with water	Twining upon *Adhatoda vasica*, near Surajkund, Tiwaripur region	[14], [22]: p. 216	
24.	*Cyperus scariosus* Br. 10810	Cyperaceae	Nagarmotha/ Chakranksha	Roots	Decoction of root is used	Shallow water bodies of study area	[22], [32]: p. 2637	
25.	*Datura metel* Linn. 10811	Solanaceae	Dhatura	Seed, Leaves, and Roots	Administered as decoction of plant parts	Frequently in waste places	[22]: p. 219, [33]	
26.	*Eclipta prostrata* (Linn.)Linn 10812	Asteraceae	Bhangraiya, Bhringaraj	Whole plant, Root and Leaves	Plant and its parts are grinded and mixed with water and given to patient	Open pastures, wet regions, along water canals	[2], [22]: p. 175	
27.	*Erythrina* *variegata* Linn. 10813	Fabaceae	Pangara	Bark	Bark paste is made in to pills and are taken till cure	Moderate sized deciduous tree planted in gardens, Near Ramgarh tal	[22]: p. 99, [28]	
28.	*Jatropha gossypifolia* Linn. 10814	Euphorbiaceae	Bhakrend	Seeds	Seeds are taken with water	Roadside weed and found at other waste ground	[22]: p. 296, [28]	
29.	*Lantana camara* Linn 10815	Verbenaceae	Ghaneri	Whole plant	Plant decoction is given	Roadsides, Wild, Wastelands and Railway colony	[22]: p. 256, [34]	
30.	*Leucas aspera* (Willd.) Link. 10816	Lamiaceae	Gopha, Drona pushpi	Leaves	Leaves used as mosquito repellent by the rural people	Weed of crop field, waste places, dry open sandy soil, Bhathat region	[14], [22]: p. 263, [41]	
31.	*Ludwigia octovalvis* (Jacq.) P.H. Raven 10817	Onagraceae	Panijalokia	Leaves	Leaf juice is used in intermittent fever	Wet places, sides of tanks	[22]: p. 141, [35]	
32.	*Magnolia grandiflora* Linn. 10818	Magnoliaceae	Andachampa	Bark	Bark is boiled in water and remaining water is given to patient	Planted in gardens, Gorakhnath temple area	[22]: p. 31	
33.	*Momordica charantia* Linn. 10819	Cucurbitaceae	Kathnim, Karavellaka	Roots and Fruits	Decoction of roots and fruits is used	Cultivated, found wild, climbing on hedges	[2], [22]: p. 149	
34.	*Murraya koenigii* (L.) Sprengel 10820	Rutaceae	Bursunga/ Gandhla	Roots	Juice of roots is given in malaria	Deciduous shrub on waste grounds along water bodies, planted in gardens	[2], [22]: p. 69, [36]: p. 472	
35.	*Nyctanthes arbor-tristis* Linn. 10821	Oleaceae	Harsingar	Leaves	250 gm leaf is boiled in ½ lit water and mix with leaf juice of *Ocium tenuiflorium*. Mix with honey, 50 ml of this for 3 days	Gardens, Bungalow, Temple and Railway colonies	[14], [22]: p. 191, [40]	
36.	*Ocimum* *sanctum* Linn. 10822	Lamiaceae	Tulsi	Roots	decoction of roots given as diaphoretic in malarial fever	Found as an escape from cultivation in moist places	[9], [22]: p. 267	
37.	*Oenanthe javanica* (BL.) DC. 10823	Apiaceae	Pan tarori	Whole Plant	Plant extract is used in mild fever	Weed in moist waste places, fruit orchards, along water channels	[32]: p. 156, [35]	
38.	*Piper longum* Linn. 10824	Piperaceae	Peeper, Peepramool	Fruits and Roots	Plant part is grinded, mixed with water and administered orally	Forest Zone of whole study area and hotter regions	[27]: p. 2128	
39.	*Pongamia pinnata* (L.) Pierre 10825	Fabaceae	Karanja	Fruit	Fruit is boiled in water and administered as decoction	Roadside, near Canals, Wasteland, Moist regions, Pharenda and Ramgarh forest	[22]: p. 108	
40.	*Putranjiva roxburghii (*Wall.) 10826	Euphorbiaceae	Putjev, Jiaputa, Putrajiva	Leaves	Leaves and stones given in decoction for cold fever	Wild, tropical and cultivated, hedge plants in gardens	[9], [27]: p. 2237, [22]: p. 299.	
41.	*Rauvolfia serpentine* (Linn.) Benth. 10827	Apocynaceae	Dhamarharua	Roots	A paste of root and black pepper is administered	Damp places, wild in forests	[14], [22]: p. 196, [28]	
42.	*Scoparia* *dulcis* Linn. 10828	Scrophularia-ceae	Mithi patti	Leaves	Two teaspoonful of leaf juice is taken twice a day	Waste places and a weed of crop fields	[22]: p. 231, [28]	
43.	*Sida rhombifolia* Linn. 10829	Malvaceae	Bariara	Roots	Boiled extract is given	Shady waste places near tals (water bodies), hedges	[22]: p. 55, [37]	
44.	*Solanum indicum* Linn. 10830	Solanaceae	Lapta Brihatti, Banbhanta	Fruits	Burnt fruits are consumed	Found as wild in whole study area	[22]: p. 222, [25]	
45.	*Stephania japonica* (Thunb.) Miers 10831	Menispermaceae	Rajpatha	Roots	Sun-dried roots powder is given orally with boiled water twice a day	Hedges on moist ground	[2]: p. 36	
46.	*Streblus asper* Lour. 10832	Moraceae	Singhor	Bark	Juice obtained from squeezed bark is taken till cure	Small evergreen tree found throughout the study area	[22]: p. 308, [28]	
47.	*Swertia chirata* Buch.Ham 10833	Gentianaceae	Chiraita, Charayatah Kirata tikta	Whole plant	Plant part is boiled in water till 75 % is evaporated and it is drink like tea	Temperate Himalayas, 4000-10000 ft, Paniara region	[27]: p. 1664	
48.	*Tinospora cordifolia* (Thunb.) Miers. 10834	Menisperma-ceae	Gurch, Giloe	Stems and Roots	Decoction of roots is given for malaria	On hedges and trees, tropical regions of study area	[2], [22]: p. 35, [36]: p. 77	
49.	*Vandellia sessiliflora* Benth. 10835	Scrophularia-ceae	Lindernia sp.Indian	Whole plant	Decoction of whole plant is used	Damp and shady places, grows with grasses	[22]: p. 229, [38]	
50.	*Vernonia cinerea* (Linn.) Less. 10836	Asteraceae	Sahdevi	Whole plant	Decoction is prepared and 2 tea spoonful is given twice a day	Weed of crop field and waste grounds	[2], [22]: p. 181	
51.	*Xanthium strumarium* Linn. 10837	Asteraceae	Lapetua	Roots	Decoction is prepared of roots of *Lapetua*	Waste places, long railway tracks and roadsides	[2], [22]: p. 183	

Beverage is also plant extract based drink which usually have low concentration than decoction

^a^Decoction is an extraction method by boiling of dissolved chemicals of medicinal plant or plant parts

### Sampling method

The survey was conducted during rainy (July–October 2013) months (Malaria epidemic season). Local population based survey sampling relying on TK system was followed. Survey locality chosen based on the study of API (Annual parasitic index) of Malaria obtained from District Malaria Office. All those areas with relatively high API were selected for the survey while respondents were chosen based on their interface with TK System and this information was obtained from a local survey while herbalists *(Vaidya/Hakim)* of the locality were given precedence. In total 85 independent respondents were chosen from Gorakhpur (22), Kushinagar (38) and Maharajganj (35) districts (Table 4).

**Table 4:** Mathematical analysis of interviewee’s response

Location	Total interviewees (M/F)	Occupation		Literacy^a^ (M/F)	Mean age^b^ (years)	Monthly income^c,d^ (INR)^e^
		Agri	Non-agri			
Gorakhpur	22 (18/4)	74 %	26 %	74.5 (79/54) %	52.7	4915.0
Kushinagar	38 (27/11)	78 %	22 %	63.7 (68/53) %	55.0	3887.0
Maharajganj	35 (28/7)	82 %	18 %	67.2 (72/48) %	48.4	4093.0
Total^f^	85 (63/22)	78 %	22 %	68.5 (73/52) %	52.0	4298.3
Details of plants						
Surveyed plants	51					
Most commonly used plants	3					
Number of families of surveyed plants	27					
Number of families with two or more plants	12					
Major plant families						
Fabaceae	7					
Asteraceae	4					
Acanthaceae	4					
Amaranthaceae	4					
Menispermaceae	3					
Most popular plants (With PK ≥ 55 %)						
*Adhatoda vasica* Nees						
*Cassia fistula* Linn.						
*Swertia chirata* Buch.Ham						
Geographical profile^g^						
Average annual rainfall		1148.3 mm				
Average relative humidity		68.33 %				
Average high temperature		30.92 °C				
Average low temperature		19.58 °C				
Average Mean Temperature		25.25 °C				
Average no of days with precipitation		44.88				
Frequency of usage of traditional knowledge		Yes (%)	No (%)			
		70 (82 %)	15 (18 %)			

^a^Calculated on weighted mean %, *M* males, *F* females

^b^Mean of ages of M and F

^c^Based on India census 2011

^d^Economics and Statistics Division, Govt. of Uttar Pradesh, India

^e^1 USD 61.11 INR

^f^Calculated on arithmetic mean  %

^g^Based on satellite imagery and Climate Research Unit (CRU) UK

### Identification of plants

Ethnobotanical data collected was observed by expert taxonomist and botanist Dr. H.B. Naithani (Senior Scientist, FRI University Dehradun, India) and taxonomic details were ascertained (Table 3). Botanical names of plants and authority were established and cross. The data obtained from ecological sampling was used to determine geographical health of any plant in terms of vulnerable, nearly extinct or under threat. This was specified in terms of PK (percentage known) = 0 or PI (plant impression) as ‘unknown’.

### Ethics statement and consent

This is to declare that the work has no completing interest. All respondents actively participated and the survey was purely participative and voluntarily in nature. Participants provided verbal informed consent to participate in this study. They were free to withdraw their information at any point of time. Participant consent was recorded on the designed sheet. The authors adhered with ISE 2006 and 2008 guidelines and all the principles laid down were followed. The entire voucher numbers of specimen collected were deposited in Forest Research Institute, Dehradun herbarium (Voucher Number 10787–10837). The work did not involve any protected area (National Park, Wildlife Sanctuary or Private Plantation). It was done on the land which can be easily accessed by the public and hence no such permission was required. The field studies did not involve any endangered or protected species. GPS co-ordinates for the study region are: Gorakhpur (Lat. 26°13′N to 27°29′N and Long. 83°05′E to 83°56′E), Kushinagar (Lat. 26°39′N to 27°15′N and Long. 83°38′E to 84°15′E) and Maharajganj (Lat. 26°59′N to 27°19′N and Long. 83°09′E to 83°45′E).

### Interview details and criteria

Eighty-five respondents from all ethnic groups comprising Tharu tribes, Hindus, Muslims were interviewed against designed format of set of questions like collection date, local name, plant image, plant location/region, plant description, method of usage of plant in malarial cases. There were 63 male and 22 female respondents from all three major localities (Table 4). No pre-test was conducted as the obtained information was cross validated with the existing TK system in literature databases (PMC etc.). Elderly (as they are TK rich) people above 50 years (mean age 52.0 years) and mainly males (74.12 %) were interviewed because of their easy availability. Samples were collected throughout the study area making each geographical representation possible (such as people residing in the area of forest, near water bodies, urban areas and rural areas). Participants were chosen based on their experience and as well as age. Plant images have been taken from the plant’s natural habitat to build digital database to facilitate the easy recognition of plant by the local people and later it is integrated with the known traditional applications of these plants as antimalarial plants. The interview was done in three modes of field, *Vaidya/Hakims* and house to house to strengthen the data collection process and at the finally it was cross checked.

### Data collection

Data on ethno-medicinal applications of plants are collected in the sequences of: Serial number, botanical name, family, vernacular/local name, part used, methodology used, plant location, and plant image. Later, collected data was referenced using surveyed literature and databases. Demographic and socio-economic survey was also done to find correlation with malaria epidemic with these factors. Additional information (Additional file 1) like age of respondents, education background, monthly income and occupation was also recorded (Table 4). It was also queried for how frequent people use TK system for malaria cure.

### Data analysis

Data obtained through ethnobotanical survey was analyses through following parameters:

1. Percentage known (PK): This is  % of people who have knowledge of usage of plant species for malaria treatment and calculated as: PK = [n/m] × 100; n = total number of positive responses and m = total number of respondents.
2. Preference ranking (PR): It is similar to the rank system used by Asase et al. [24]. Here, plants were ranked as per their effectiveness towards treatment. Rank 1, 2 and 3 were assigned where rank 1 means highly effective in treatment while rank 3 means least effective.
3. Plant impression (PI): It is the index indicating how a plant is perceived in the locality. PI is comprised of four categories as good, fair, poor and unknown. PI is ‘Good’ if PK ≥ 50 %; ‘Fair’ if 30 ≤ PK < 50 %; ‘Poor’ if 0 < PK < 30 %; and ‘Unknown’ if PK = 0 %. PK = 0 means plant is unknown among community people but found in the field and also available in the literatures.
4. Graphical analysis: Pie charts have been drawn to assess the most frequent part of plant usage in TK system of antimalarial plants (Graph 1), major plant families (Graph 2), response of people and patients (Graph 3) and folklore (Graph 4).
5. Analysis of the interviewee response [17] has been presented (Tables 2 and 4) to include observations related to geographical profile of study area, literacy, monthly income, occupation etc.

## Results

Traditional knowledge system of antimalarial plants: Compilation of all antimalarial plants prevalent in the region (Table 3).

**Fig. 3:**
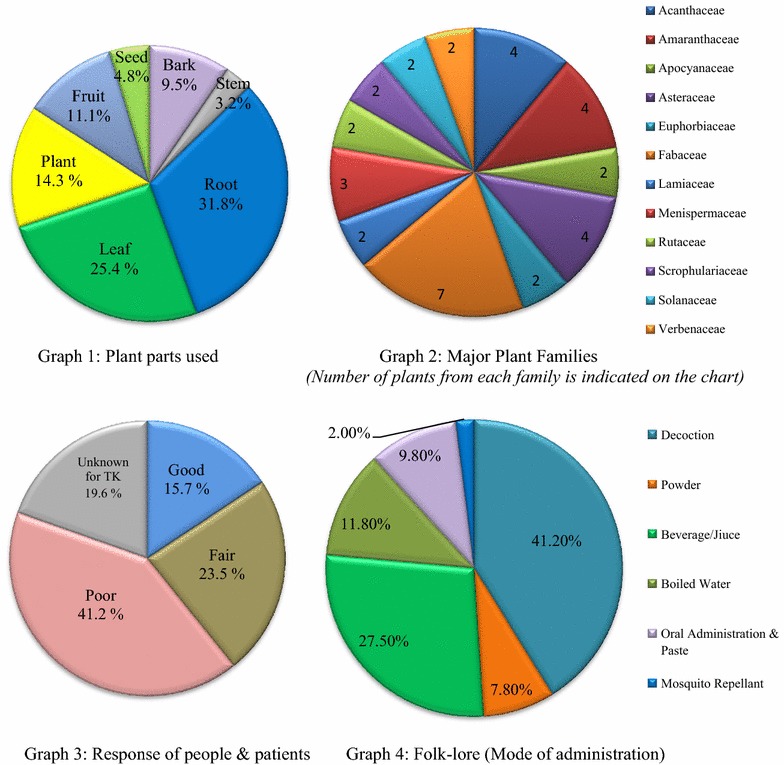
Graphical analysis

Graphical analysis (Fig. 3): Four pie charts are drawn and results are:

- Roots are mainly (31.8 %) used towards malaria cure (Graph1).
- As many as 12 plant families have more than one plant in the region (Graph 2) and Fabaceae family makes highest influence of 7 plants in the list (Table 3).
- Response of people and patients was analysed. It was found that 41.2 % responses are ‘poor’ while 19.6 % people are unknown for any TK Based system in the region (Graph 3).
- In 41.2 % cases plant part used as decoction followed by plant usage as juices or in some form of beverage# (27.5 %) and plants are also used as mosquito repellent (2 %) (Graph 4).

Plant identification and people’s preferences:

- Considering people’s perception there are 11 plants reported to be ‘unknown’ to the community people against malaria treatment while 8 plants are ‘Good’, 12 plants are ‘Fair’ and 21 plants are ‘Poor’ in their efficacy.
- Only 7 plants were reported during all three modes of interview conducted.
- *Cassia fistula* has got highest number of positive response (n = 51) among community people, followed by *Adhatoda vasica* (n = 49) and *Swertia chirata* (n = 48). All these plants are most popular antimalarial plants.

Socio-economic and ethnographic results:

- 97.48 % population is agrarian, mean age of respondents is 52.0 years, total literacy is 68.5 %, monthly income is 4298.3 INR (~70.3 USD), and Gender (M/F) respondents were (78/22) %. TK system usage is among 82 % of population but they also rely on modern method of allopathic treatment when TKS becomes ineffective.

## Discussion

In the study area two species namely Nagarmotha (*Cyperus scariosus*) and Goma or Drona pushpi (*Leucas aspera*) are at the verge of extinction mainly due to the change in water reservoir structure i.e. because of loss of water bodies like ponds and other reservoirs for the former and because of change in land-use pattern for the latter. The ethnoecology of the study region does not produce the scintillating picture. Further, due to ruthless exploitation, many important medicinal plants species are becoming rare and some of them are critically endangered as per the IUCN (International Union for Conservation of Nature and Natural resource), Switzerland red list criteria. It is estimated that 10 % of all plant species are currently endangered in India [39].

It is found that 51 plant species out of various plants studied in the region belong to 27 families are used by native people malaria treatment (Table 3) and out of these families studied certain family of plants like, Fabaceae (7), Acanthaceae (4), Amaranthaceae (4), Asteraceae (4), and Menispermaceae (3) are prevalent in the region. A commendable work has been done by Duthie for the flora of Flora of Upper Gangetic Plains [21] and by Srivastava for the Flora Gorakhpurensis [22]. Some plants like *Cosmos sulphurous, Piper longum and Swertia chirata* were found in the study area which missed its place in the Srivasatava’s work and also these are not listed in Duthie’s work. These plants might have grown up in the area during the recent decades or may have come through migrations.

Spatial distribution of antimalarial plants found in the study area including types of habitat is supplied in Table 3. Plant diaspora usually found in the tree/forest, open pastures, river and water body regions; while some are found in some specific locations like Lahladpur, Surajkund, Tiwaripur, Ramgarh Tal, Gorakhnath Temple, Pharenda (forest), Ramgarh (forest), Paniara, Bhathat areas whereas and some are found vaguely throughout the region. Further, there are few plants which are found in fruit orchards, waste/open areas and along railway tracks. Some are distributed in moist waste places, crop fields and along roadside and some are in marshy lands, moist places, garden and temples (in settlements) and near *nallas*/canals.

The region falls in low socio economic zones with monthly income ~70.3 USD while population is agrarian (97.48 %) and hence their obvious dependency for malaria cure is TKS. It is also found that malaria incidences are relatively higher in the socio-economic inferior regions. There are sizeable amount of *Vaidya/Hakims* (Experts in TKS based applications) who derive their raw material from nearby forests, railway tracks, fruit orchards or from target plantations. The region has 1,436,878 as total households in which 87.98 % is rural while merely 12.02 % is urban and demographic divide lies with 51.25 % males while 48.75 % females in the entire study area [20].

In drawing Pie Chart for plant parts used; rationale used is total number of plants with a particular plant part used is divided by total plants in the study area and multiplied by 100 to compute its  %. It is found that among collected plants mostly (31.75 %) roots are used for malaria treatment followed by leaf (25.40 %) and the least used part of plant is stem (3.17 %) preceded by seeds (4.76 %) (Graph 1). Details of most widely plant parts used is necessary to ascertain the significance of that particular part and to establish the coherence of the work with other similar works e.g. for the medicinal plants of BHU region of Varanasi UP [2]. It has been established that for that given region 30 % of preparations are derived from roots which is maximum in plant parts used. In another similar work of Shankar et al. [13] on antimalarial plants of northeast India, frequently used plant parts were roots (31 %), after leaves (33 %). It can be established that in most of the cases root is most frequently used towards medicinal purposes and in some cases leaves are also widely used. Thus, there is a need of expertise for extraction of artemisinin or quinine alkaloid compounds preferably from the roots and leaves.

There are 12 plant families including Acanthaceae, Amaranthaceae, Apocyanaceae, Asteraceae, Euphorbiaceae, Fabaceae, Lamiaceae, Menispermaceae, Rutaceae, Scrophulariaceae, Solanaceae and Verbenaceae that have more than one plant in the region (Graph 2) while Fabaceae makes highest contribution of 7 plants namely *Abrus precatorius, Acacia farnesiana, Bauhinia variegate, Caesalpinia crista, Cassia fistula, Erythrina variegate* and *Pongamia pinnata* (Table 3). Response of these plants among people and patients was upright and it had been acting as good alternative to modern means of medication and had been catering to the need of millions from the ancient times. Folklore (mode of administration) of plant parts is decoction (41.2 %) where plant parts are boiled in water and water is evaporated to extend that plant part makes thick syrupy liquid. It is an excellent way to prepare herb with an awful taste. Decoctions of roots and barks are very often prepared while decoctions of leaves, flowers, or seeds are rarely prepared.

The interview was trifurcated in three categories of field, *Vaidya/Hakims* and house to house to understand the method of plant identification and people perception (Table 2). It was found that as many as seven plants were obtained through all three interview modes. Three indices PK, PR and PI are used to understand perception of plants among community people. Total number of positive responses (n) were collected and combined for all 51 reported plants. PK signifies popularity of a plant as antimalarial. If PK > 55 %; such plants are considered as most popular plants. For 11 plants, PK = 0 was reported that signifies plant is unknown in the locality among people, during field survey they were found and such plants also found space in literature and journals. Plants with PK = 0 are highly important and further work has to be done to enhance its PI which will lead to increased plant’s medicinal utility. These are the plants which have least importance among communities and these are ecologically available for future potential application. These plants are not known due to over-dependence on plants with PK > 55, in the study area.

PR has been assigned to each plant either as 1, 2 or 3 depending on people perception as well as research findings. 1 signifies for highly effective in treatment while 3 for least effective in treatment. There are 19 plants with PR as 1 i.e. They are rated as effective in malaria treatment. It is observed that indices like PR and PI are in resonance for most of the plants; higher is PR (PR = 1) higher is PI (Good) and vice versa and runs parallel. 21 plants have ‘Poor’ PI (41.2 %). It is because of its inefficacy towards fever, intermittent fever or malaria cure and hence these plants are least preferred by the local communities. IUCN status of plants with ‘fair’ and ‘good’ impression (PK > 30 %) towards malaria cure is tabulated to understand geographical health of the plant as per the red list criteria (Table 5).

**Table 5:** IUCN status for frequently used plants towards malaria cure

S. no	Plant species	IUCN status	S. no	Plant species	IUCN status
1	*Abrus precatorius*	Not threatened	11	*Cassia fistula* Linn	Not yet been assessed
2	*Achyranthes aspera*	Not yet assessed	12	*Cuscuta reflexa* Roxb	Not yet been assessed
3	*Adhatoda vasica*	Not yet assessed	13	*Cyperus scariosus*	Not yet been assessed
4	*Alstonia scholaris*	Least concerned	14	*Leucas aspera*	Not yet been assessed
5	*Andrographis paniculata*	Not yet assessed	15	*Ocimum sanctum*	Not yet been assessed
6	*Azardirachta indica*	Not yet assessed	16	*Piper longum*	Not yet been assessed
7	*Bauhinia variegata*	Not yet assessed	17	*Pongamia pinnata*	Least concerned
8	*Boerhaavia diffusa*	Not yet assessed	18	*Putranjiva roxburghii*	Not yet been assessed
9	*Caesalpinia crista*	Not Threatened	19	*Swertia chirata*	Not yet been assessed
10	*Carica papaya*	Not yet assessed	20	*Tinospora cordifolia*	Not yet been assessed

Based on IUCN red list criteria

Having life in close vicinity of the nature, traditional societies have acquired unique knowledge about the use of flora and fauna in wild, most of which are still unknown to the common masses. The research findings can be used by both scientific community and common rural/tribal people for bio-prospecting of these natural resources sustainably. The former can extract the tables obtained in the research work to obtain a suitable plant for finding a lead molecule towards a drug discovery project; while the latter can use Table 3 information to meet the local demand for malaria cure. Plant image, methodology and plant location can be seen together to bring malaria cure measures in friendly manner for local communities at their door step. This will be much useful for the developing countries like India where the modern medical facilities have still not penetrated down deeper. It provides sustainable alternate to the existing system of malaria medication. Findings of such works should be made open and unrestricted for its elaborated application to benefit the public at large. The work has also included many plants that are not widely used and are not popular in the study area. This widens the scope of extraction of other non-traditional plants in cases of poor ethnoecology especially during extinction or vulnerability of the traditional plants because of over harvesting and over exploitation of these medicinal plants.

## Conclusion

The study region has monsoonal rainfall with numerous rivers emanating from Himalayas and low gradient leads to stagnation of water that makes it favourable for vector borne diseases. TK coupled with information technology makes the study highly useful for basic amenity stressed population. The lack of ecology-related knowledge and awareness which is evident from low socio-economic profile of the study region gives signs of over harvesting of plants. This is reinforced by the observed poor geographical health of plants as some plants are found vulnerable; some has become rare, while some are nearly extinct. It further widens the horizon of socio-economic based utility of this kind of research work. The research work fully addresses the issue of making public the availability of information of antimalarial plants for its cheapest and sustainable harvesting for malaria cure locally and it opens gates for numerous opportunities of public health at large.

The work carried out revealed the plants recorded are highly valuable for antimalarial application and in future, bio-prospecting projects can be further initiated for sustainable harvesting towards developing antimalarial drug for curing malaria at large. It would help researchers to find out suitable lead molecules with antimalarial activity towards drug discovery. The study provides ample ground to believe that the traditional medicinal system practice using native medicinal plants is alive and well functioning in the selected area. Many communities use antimalarial plant parts and whole plant for their primary healthcare. It is primarily due to lack of modern medicines, medications, self-reliance on herbs, poor economic condition and more importantly faiths in TK System. The treatment of malaria with plants and plant parts causes little or no side effects and also it is very cheap and affordable. Some plants are nearly extinct in the region, the reason being change in land use pattern and shrinking of water bodies along with over harvesting of herbs. The bio-depletion of these antimalarial plants is due to the burgeoning population and unscientific management of the natural resources.

The work has highlighted the fact that some species with medicinal applications are not ethnoecologically rich. People residing in the study area are not aware that some of the medicinal plants are becoming extinct gradually. The current work of listing these antimalarial plants would assist people in identifying ecologically sensitive species and hence adoption of regeneration technique for that particular plant species, thus it supports health and economy of the community. There exists alarming need to conserve and protect these important species for sustainable harvesting. A serious endeavour has to be taken to save these natural resources for the generations to come. Study area in the work happens to be least developed part of one of the poorest State in India. Thus, working for low socio-economic profile region will certainly be useful at larger scale and will bear extended benefits when the work will be thoroughly evaluated.

## Future work

Exhaustive research should be done on comparative analysis of different antimalarial plants to comprehend if the local evaluation of the effectiveness of the different species can be scientifically authenticated. Listed medicinal plants can be potentially used for extracting antimalarial plants content for further chemical extraction and its analysis as a potential constituent of malaria drug can be established. The plants can be further studied across its family to establish antimalarial activity relationship of various families and correlation based study can also be done to generate coefficient of correlation for a particular plant family as an antimalarial. The present findings can be extended to chalk out a complete roadmap of geographical health of antimalarial plants in terms of IUCN criteria of red list and for detailed ethnoecology work.

## Additional file

10.1186/s13104-015-1827-z Survey format.
