# Supplementary material for: Ethnobotanical perspective of antimalarial plants: traditional knowledge based study
Source: BMC Res Notes. 2016 Feb 4;9:67. doi: 10.1186/s13104-015-1827-z (PMC4743172; doi:10.1186/s13104-015-1827-z)
Supplement: Supplementary file 1 — 10.1186/s13104-015-1827-z Survey format. [file 13104_2015_1827_MOESM1_ESM.docx]

**SURVEY ^#^ FORMAT**

1. Respondent No:
2. Date of Survey:
3. Local Name/s of plant used for malaria:
4. Whether s/he is beneficiary of TKS ^*^? : Yes/No
5. How often they use it for treatment:
6. How effective is TK method:
7. Dependency on allopaths:
8. Method (folklore) they use:
9. Sex (Male/Female):
10. Age:
11. Religion:
12. Are you scheduled tribe? Yes/No
13. Educational Qualification:
14. Occupation:
15. Monthly Income:

**Additional Work (Outside survey work):**

1. Digital snap of plant part:
2. Taxonomic details:
   1. Family
   2. Botanical name:
   3. Detailed folklore:
   4. Local name:
   5. Plant Part used:
3. Plant Location:
4. Lat/Long co-ordinates:
5. References:

TKS ^*^ : Traditional Knowledge System.

^#^ Survey sheet was in consonance with umbrella project of OSDD initiated by CSIR in India. Survey sheet was accepted by several discussions and then it was conducted.

Personal/demographic information was obtained from participants:

- Personal/demographic information related to their age, sex, religion, ethnicity, occupation, monthly income, local name of plant they use, method (folklore), extent of dependency on TKS for malaria treatment, beneficiary of TKS or not and dependency on allopath was obtained from the participants/respondents.
